# Supplementary material for: Updated site concordance factors minimize effects of homoplasy and taxon sampling
Source: Bioinformatics. 2022 Nov 16;39(1):btac741. doi: 10.1093/bioinformatics/btac741 (PMC9805551; doi:10.1093/bioinformatics/btac741)
Supplement: btac741_Supplementary_Data [file btac741_supplementary_data.docx]

# Formulation of the updated sCF

Given a sampled state-quartet at a particular site, we say it is decisive if it is parsimony informative. For decisive sites, the state-quartet is concordant with **x** if it supports bipartition $\{a,b\}|\{c,d\}$ (i.e. it matches the bipartition in the species tree). It is discordant if it supports either bipartition $\{a,c\}|\{b,d\}$or $\{a,d\}|\{b,c\}$. The concordance factor (CF) can then be calculated for a single sample of states in a quartet, *q*, for each of *j* sites in an alignment:

$$\begin{aligned} {CF}_{q}\left( \mathbf{x} \right)=\frac{\left| \left\{ j:j is concordant with \mathbf{x} \right\} \right|}{\left| \left\{ j:j is decisive with \mathbf{x} \right\} \right|},\#\left( 1 \right) \end{aligned}$$

where the operator $\left| j \right|$ denotes the count of sites that meet the given criterion. To account for uncertainty in ancestral states, the sCF for branch **x** is calculated by averaging over *m* sampled quartets:

$\begin{aligned} sCF\left( \mathbf{x} \right)=\frac{1}{m}\sum_{q=1}^{m} {CF}_{q}\left( \mathbf{x} \right).\#\left( 2 \right) \end{aligned}$

If there is one tip in a clade, the observed state at the tip is used. In the previous version of the statistic, we sampled observed states from the tips of taxa contained within clades $A,B, C, and D$. Therefore, the updated sCF is equivalent to the previous version for a four-taxon tree, with one tip in each clade.

# Simulation settings

The tree with 20 taxa in clade *E* is given below in Newick format. For simulations with fewer taxa in clade *E*, we simply removed them from this string.

((((A:0.3,B:0.3):0.7,C:1):2,D:3):17,(E1:19.0,(E2:18.0,(E3:17.0,(E4:16.0,(E5:15.0,(E6:14.0,(E7:13.0,(E8:12.0,(E9:11.0,(E10:10.0,(E11:9.0,(E12:8.0,(E13:7.0,(E14:6.0,(E15:5.0,(E16:4.0,(E17:3.0,(E18:2.0,(E19:1.0,E20:1.0):1.0):1.0):1.0):1.0):1.0):1.0):1.0):1.0):1.0):1.0):1.0):1.0):1.0):1.0):1.0):1.0):1.0):1.0));

When there are no taxa in clade *E*, we used the following four-taxa tree:

(((A:0.3,B:0.3):0.7,C:1):2,D:3);

For each simulation condition, 1,000,000 gene trees were simulated in *ms* (Hudson 2002), and sequences were generated with *Seq-Gen* (Rambaut and Grassly 1997) setting the population mutation parameter *θ*=0.05. The commands used to generate data for the 20-taxa case in clade *E* are:

./msdir/ms 24 1000000 -T -I 24 1 1 1 1 1 1 1 1 1 1 1 1 1 1 1 1 1 1 1 1 1 1 1 1 -ej .5 24 23 -ej 1.0 23 22 -ej 1.5 22 21 -ej 2.0 21 20 -ej 2.5 20 19 -ej 3.0 19 18 -ej 3.5 18 17 -ej 4.0 17 16 -ej 4.5 16 15 -ej 5.0 15 14 -ej 5.5 14 13 -ej 6.0 13 12 -ej 6.5 12 11 -ej 7.0 11 10 -ej 7.5 10 9 -ej 8.0 9 8 -ej 8.5 8 7 -ej 9.0 7 6 -ej 9.5 6 5 -ej 10 4 5 -ej 1.5 3 4 -ej 0.5 2 3 -ej 0.15 1 2 | tail -n +4 | grep -v // > ms_sims.txt

./seq-gen/source/seq-gen -m HKY -l 1 -n 1 -s 0.05 ms_sims.txt seqgen_sims.txt

References

Hudson, R. R. (2002). Generating samples under a Wright-Fisher neutral model of genetic variation. *Bioinformatics*, **18**, 337-338.

Rambaut, A. and N. C. Grassly (1997). Seq-Gen: an application for the Monte Carlo simulation of DNA sequence evolution along phylogenetic trees. *CABIOS*, **13**, 235-238.
